# Dual inhibition of ERG11 and CDR2 in drug-resistant *Candida albicans* by Indian phytochemicals: a combined *in silico–in vitro* approach

**DOI:** 10.3389/fphar.2025.1687392

**Published:** 2026-01-14

**Authors:** Akshay Kisan Mundhe, Reena Rajkumari

**Affiliations:** Department of Integrative Biology, School of Bio Sciences and Technology, Vellore Institute of Technology, Vellore, Tamil Nadu, India

**Keywords:** AlphaFold, antifungal resistance, cryptococcus, molecular docking, molecular dynamics simulation, phytochemical library, structure-based drug design, trichosporon

## Abstract

Invasive candidiasis caused by drug-resistant *Candida albicans* is an escalating global health concern due to the declining efficacy of conventional antifungal therapies. In this study, Indian medicinal phytochemicals were investigated as potential dual inhibitors of the key resistance determinants ERG11 (lanosterol 14α-demethylase) and CDR2 (efflux protein *Candida* drug resistance 2) of *C. albicans* using a combined in *silico–in vitro* approach. Structure-based virtual screening of 17,967 phytochemicals from the IMPPAT (Indian Medicinal Plants, Phytochemistry and Therapeutics) database identified eleven high-affinity candidates. Five lead compounds—dalspinin-7-O-β-D-galactopyranoside, glycyrol, isokurarinone, licoflavone A, and liquiritin—exhibited strong binding toward ERG11 (−9.2 to −9.5 kcal/mol), outperforming fluconazole (−7.3 kcal/mol). Except for isokurarinone, all compounds also demonstrated effective binding to CDR2, indicating dual-target potential. Molecular dynamics simulations (100 ns) confirmed the structural stability of ERG11–ligand complexes, with liquiritin and glycyrol showing the most persistent interactions. Phytochemicals were experimentally confirmed from *Glycyrrhiza glabra* extracts using HR-LCMS and exhibited concentration-dependent antifungal activity against multiple drug-resistant *Candida* and non-*Candida* yeast pathogens *in vitro*. Collectively, these findings demonstrate the promising antifungal potential of Indian phytochemicals as dual ERG11–CDR2 inhibitors. These results provide a strong basis for developing phytochemical-based antifungal leads for future therapeutic applications.

## 1 Introduction

### 1.1 Antifungal resistance as a global therapeutic challenge

Invasive candidiasis, predominantly caused by *Candida albicans*, remains a major cause of morbidity and mortality among immunocompromised patients worldwide (Ford et al., 2015; Tsay et al., 2020; Falagas et al., 2010; Brown et al., 2012; Mazi et al., 2022). Despite the availability of azoles, polyenes, echinocandins, and flucytosine, the clinical management of candidiasis is increasingly compromised by the rapid emergence of antifungal resistance (Whaley et al., 2017; Davari et al., 2020; Carolus et al., 2020; Aldardeer et al., 2020; Arendrup and Patterson, 2017). Azole resistance, in particular, has become widespread due to the prolonged and often empirical use of fluconazole in clinical and prophylactic settings (Whaley et al., 2017; Arendrup and Patterson, 2017; Arendrup, 2014). Drug-resistant *C. albicans* strains now account for a substantial proportion of treatment failures in hospital-acquired infections (Brown et al., 2012; Mazi et al., 2022; Aldardeer et al., 2020; Arendrup and Patterson, 2017). The limited number of antifungal drug classes, host toxicity of polyenes (Carolus et al., 2020), rising echinocandin resistance (Davari et al., 2020), and high cost of newer antifungal agents collectively underscore the urgent need for alternative, resistance-breaking therapeutic strategies (Arendrup and Patterson, 2017; Arendrup, 2014; Cowen et al., 2015; Perlin, 2015).

### 1.2 Molecular rationale for dual targeting of ERG11 and CDR2

Azole resistance in *C. albicans* is primarily mediated through two interlinked molecular mechanisms: (i) alterations in ERG11 (lanosterol 14α-demethylase), and (ii) overexpression of ATP-binding cassette (ABC) efflux transporters, particularly CDR2 (efflux protein *Candida* drug resistance 2) (Cowen et al., 2015; Prasad and Rawal, 2014; White et al., 2002). ERG11 encodes lanosterol 14α-demethylase, a cytochrome P450 enzyme essential for ergosterol biosynthesis and fungal membrane integrity (Lepesheva and Waterman, 2007). Mutations in ERG11 reduce azole binding affinity, while ERG11 overexpression compensates for enzymatic inhibition, enabling sustained ergosterol production under drug pressure (Flowers et al., 2015; Dunkel et al., 2008; Singh et al., 2016).

Simultaneously, CDR2 functions as a high-capacity ATP-dependent efflux pump that actively exports azole molecules from fungal cells, thereby lowering intracellular drug concentration below fungistatic levels (Prasad and Rawal, 2014; Sanglard et al., 1999; Prasad et al., 2015). Clinical isolates frequently exhibit the simultaneous upregulation of ERG11 and CDR2, demonstrating that azole resistance arises from coordinated multi-gene resistance networks rather than single-gene alterations (Cowen et al., 2015; Prasad and Rawal, 2014; White et al., 2002; Vermitsky and Edlind, 2004).

Although combination therapy using an ERG11 inhibitor together with an efflux pump inhibitor has been explored, such strategies are often limited by increased toxicity, pharmacokinetic incompatibility, drug–drug interactions, and rapid emergence of multidrug tolerance (Cowen et al., 2015; Perlin, 2015; Prasad and Rawal, 2014). From a pharmacological and translational perspective, a single small molecule capable of concurrently inhibiting both ERG11 and CDR2 offers substantial advantages over combination therapy, including improved bioavailability, simplified dosing, reduced toxicity risk, and diminished likelihood of resistance development (Cowen et al., 2015; Prasad and Rawal, 2014; Domingos and Batzelladine, 2024). Therefore, the identification of a single compound with intrinsic dual-inhibitory capacity against both ERG11 and CDR2 represents a mechanistically superior and clinically viable antifungal strategy.

### 1.3 Phytochemicals as multi-target antifungal agents and study rationale

Medicinal plant-derived phytochemicals represent a vast and chemically diverse reservoir of bioactive compounds with inherent multi-target properties. Flavonoids, coumarins, alkaloids, and phenolic glycosides are well documented to inhibit cytochrome P450 enzymes, disrupt fungal membrane integrity, interfere with ABC transporter activity, and induce oxidative stress–mediated fungal apoptosis (Martins et al., 2016; Zang, 2020; Cushnie and Lamb, 2005; de Freitas et al., 2019; Rocha et al., 2019; Srivastava et al., 2022). Unlike conventional synthetic antifungal drugs that are optimized for single-target specificity, phytochemicals exhibit natural polypharmacology, making them particularly suitable for single-molecule dual-target inhibition strategies (Cushnie and Lamb, 2005; de Freitas et al., 2019; Rocha et al., 2019; Srivastava et al., 2022). *Glycyrrhiza glabra* is a well-known medicinal plant with proven broad-spectrum antimicrobial and antifungal activity (Martins et al., 2016; Li et al., 2021; Mamedov and Egamberdieva, 2019). Several of its flavonoids and coumarins have demonstrated anti-*Candida* effects; however, their simultaneous molecular interactions with ERG11 and CDR2 have not been previously elucidated at the structural and mechanistic level (Martins et al., 2016; Zang, 2020; Li et al., 2021). Furthermore, most available studies focus on isolated antifungal effects, whereas systematic large-scale screening of Indian medicinal phytochemicals for single-compound dual inhibition of ergosterol biosynthesis and drug efflux remains largely unexplored (Domingos and Batzelladine, 2024; Dhanasekaran et al., 2024; Maurya and Mishra, 2022; Benjamin et al., 2023; Dantas et al., 2025).

In this context, the current work was especially formulated to find individual Indian phytochemicals that can function as single-molecule dual inhibitors of ERG11 and CDR2 in drug-resistant *C. albicans*, instead of utilizing combination-based treatment strategies. This study presents a mechanistically sound and translationally pertinent paradigm for the development of phytochemical-based dual-target antifungal drugs by the integration of large-scale *in silico* screening, molecular dynamics simulation, phytochemical confirmation by HR-LCMS (High-Resolution Liquid Chromatography-Mass Spectrometry), and *in vitro* antifungal validation.

## 2 Materials and methodology

### 2.1 Computational methodology

#### 2.1.1 Preparation of proteins

**FIGURE 1:**
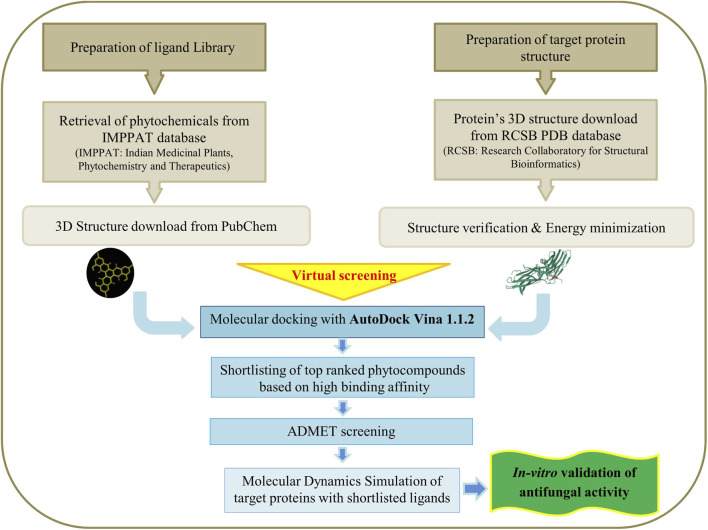
*In-silico* screening of a phytochemical library derived from the IMPPAT database to identify compounds with potential antifungal activity.

The three-dimensional models of the target proteins, ERG11 and CDR2, were obtained from AlphaFold with codes AF: P10613 and AF: P78595, respectively, in PDB (*Protein Data Bank*) format. The protein constructs were initially scrutinized for any missing residues. Energy reduction was subsequently conducted with the AMBER FF99 force field *(parameter set used in the sophisticated open-source AMBER software package for molecular dynamics simulations of biological macromolecules including proteins and nucleic acids)* in UCSF (University of California, San Francisco) Chimera edition 1.17.3 (Pettersen et al., 2004). Furthermore, Ramachandran plots were utilized to illustrate the rotational angles of these protein backbones, emphasizing areas of elevated energy and confirming the structural integrity (Ramachandran et al., 1963). The optimized protein structures were then imported into AutoDock Vina 1.1.2 (Dhanasekaran et al., 2024). In this program, polar atomic hydrogen was introduced, non-polar hydrogens were consolidated, and then Kollman charges were allocated. The finalized protein structures were stored in PDBQT (Protein Data Bank, Partial Charge (Q), & Atom Type (T)) format for the computerized screening of phytochemicals obtained from the IMPPAT library using AutoDock Vina (Figure 1).

#### 2.1.2 Preparation of ligands

Phytochemicals utilized for the computational screening were obtained from the IMPPAT (Indian Medicinal Plants, Phytochemistry and Therapeutics) database (https://cb.imsc.res.in/imppat) (Vivek-Ananth et al., 2023). Exluding the primary metabolites, a complete, unique set of 17,967 phytochemical agonists, eliminating closely repetitive metabolites, were chosen and downloaded from the PubChem database, all possessing known three-dimensional structures (Kim et al., 2023). The compounds were processed utilizing OpenBabel, which includes addition of hydrogen atoms and conducting energy minimization via the conjugate gradient refinement procedure utilizing the MMFF94 force field (Halgren, 1996). The reduced structures were archived in.mol2 format (O'Boyle, 2011). Thereafter, the.mol2 files were transformed into.pdbqt format with the script mol2s_to_pdbqts.sh (Li and Shah, 2017) (Figure 1).

#### 2.1.3 Prediction of active sites

The active sites of the target molecules were identified utilizing the CASTp (Computed Atlas of Surface Topography of Proteins) web server (http://sts.bioe.uic.edu/castp/calculation.html), which detects topological cavities and binding nooks on protein surfaces (Binkowski et al., 2003). Literature data was used to validate the functional significance of the anticipated amino acid residues mediating agonist interaction.

#### 2.1.4 Optimization of molecular docking

 Molecular docking of phytochemical ligands to the target protein was conducted with AutoDock Vina version 1.1.2 (Trott and Olson, 2010) (Figure 1). Distinct docking grids were defined for each protein based on their respective active or functional regions. For ERG11, the docking grid was centered at X = –44.389, Y = –12.556, Z = 21.972 with grid dimensions of 70 × 50 × 50 Å (Table 1). In contrast, docking against CDR2 employed a larger grid centered at X = –9.364, Y = –7.255, Z = –6.063 with dimensions of 100 × 100 × 100 Å (Table 2), reflecting the broader binding cavity of this efflux transporter. 

**TABLE 1:** Configuration used for molecular docking for ERG11.

Docking dataset	center_x	center_y	center_z	size_x	size_y	size_z	energy_range	Exhaustiveness	num_modes
For total phytochemicals	−44.389	−12.556	21.972	70	50	50	3	8	9
For top 500 phytochemicals	−44.389	−12.556	21.972	70	50	50	3	32	20

**TABLE 2:** Configuration used for molecular docking for CDR2.

Phytochemical set	center_x	center_y	center_z	size_x	size_y	size_z	energy_range	Exhaustiveness	num_modes
For total phytochemicals	−9.364	−7.255	−6.063	100	100	100	3	8	9
For top 500 phytochemicals	−9.364	−7.255	−6.063	100	100	100	3	32	20

During the initial high-throughput virtual screening of 17,967 phytochemicals against both proteins, an energy range of 3 kcal/mol was applied using standard AutoDock Vina parameters (exhaustiveness = 8, num_modes = 9) to enable efficient large-scale docking. Based on binding affinity scores, the top 500 ligands for each target protein were shortlisted for further refinement. Subsequent docking of these selected compounds was carried out with increased stringency by setting the exhaustiveness to 32 and the number of output binding modes to 20, thereby improving conformational sampling and the accuracy of predicted binding poses. 

Each docking run generated two output files per ligand: a log file summarizing docking statistics and a PDBQT file containing multiple predicted binding conformations along with their corresponding binding energies (kcal/mol). AutoDock Vina produced up to 20 ranked conformations per ligand, ordered according to predicted binding affinity, with the lowest binding energy conformation considered the most favorable. Visualization and interaction analysis of the docked ERG11–ligand and CDR2–ligand complexes were performed using UCSF Chimera version 1.17.3 (Pettersen et al., 2004) and Discovery Studio 2021.

#### 2.1.5 Drug likeness and ADME/toxicity prediction

SwissADME (ADME: Absorption, Distribution, Metabolism, and Excretion) (http://www.swissadme.ch/index.php) is a publicly accessible online platform that provides rapid and dependable predictions for several criteria, covering pharmacokinetics, drug-likeness, physicochemical properties, and medicinal chemistry compliance. It utilizes specialized instruments such the BOILED Egg model, Bioavailability Radar, and iLOGP for thorough profiling (Daina et al., 2017). This study evaluated the drug-likeness features of the top 11 ligands found by molecular docking utilizing the SwissADME system (Daina et al., 2017). Assessments encompassed physicochemical characteristics, pharmacokinetic profiles, pharmacodynamic attributes, and overall medicinal chemical viability. Furthermore, ADMETlab 2.0 (https://admetlab3.scbdd.com/server/evaluationCal) was utilized to forecast toxicity assessments and bioavailability metrics of the lead drugs (Dhanasekaran et al., 2024; Daina et al., 2017; Xiong et al., 2021).

#### 2.1.6 Molecular dynamics simulation

Subsequent to the molecular docking data, molecular dynamics (MD) simulations were performed on ERG11, both in its apo forms and in association alongside the leading five ligands that demonstrated the lowest binding energy. The simulations were conducted with GROMACS 2022.5 with the CHARMM force field for duration of 100 nanoseconds (http://www.gromacs.org/) (Dhanasekaran et al., 2024; Schüttelkopf and Van Aalten, 2004; MacKerell et al., 1998). The system configuration was accomplished using the steepest descent approach, which successfully minimized the vacuum energy over 2000 iterations. A cubic periodic box was employed to solvate the complex molecule using the TIP3P (Transferable Intermolecular Potential with 3 Points) water model, guaranteeing adequate cushioning of water molecules around the protein. The system was adjusted to a physiological salt content of 0.15 M by adding sufficient Na+ and Cl− counter ions. Subsequent to the NVT (stands for constant Number, Volume, and Temperature ensemble) and NPT (stands for constant Number, Pressure, and Temperature ensemble) simulations, each enduring 1,000 picoseconds, the system was brought to equilibrium to guarantee ideal temperature, pressure, and density. The system configuration was executed in accordance with the protocols for dynamic simulation. Subsequent to the NPT equilibration phase, the ensemble performed a concluding simulation run of 100 ns (Dhanasekaran et al., 2024; Prakash et al., 2020). In the last phase, the trajectory was examined utilizing several GROMACS analytic methods. The structural and dynamic behaviors of the apo protein and its complexes with the top-ranked phytochemicals, along with the reference drug miconazole, were analyzed using standard GROMACS tools. The calculations for RMSD, RMSF, radius of gyration (Rg), and solvent-accessible surface area (SASA) were performed using the commands gmx rmsd, gmx rmsf, gmx gyrate, and gmx sasa, respectively. Hydrogen-bond interactions in the protein–ligand complexes were evaluated using the gmx hbond utility to characterize stabilizing contacts between the ligand and protein. All XVG output files were visualized and analyzed using XMGrace v5.1.25, facilitating a comparative assessment of conformational stability, flexibility, compactness, and solvent exposure among the simulated systems (Dhanasekaran et al., 2024; Maurya and Mishra, 2022; Benjamin et al., 2023).

### 2.2 Experimental methodology

#### 2.2.1 Plant material and extraction of phytochemicals

Among the five selected phytochemicals, glycyrol, licoflavone A, and liquiritin were predicted to be present in *Glycyrrhiza glabra* as per the IMPPAT database. Clean, dried root sticks of *G. glabra* were procured from a local authenticated herbal supplier and finely powdered to enhance extraction efficiency. The powdered material was subjected to solvent extraction using a 1:8 (w/v) ratio of 75% ethanol and 75% methanol, following standard protocols for efficient recovery of flavonoids and coumarins (Laboukhi-Khorsi et al., 2017).

**FIGURE 2:**
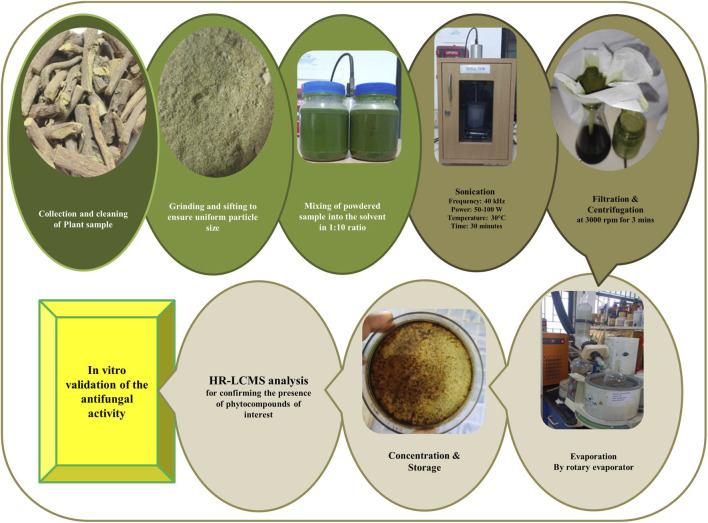
Extraction and analytical evaluation of *Glycyrrhiza glabra* phytochemicals, confirming the presence of liquiritin, licoflavone A, and glycyrol, followed by *in-vitro* evaluation of their antifungal activity.

Ultrasonic-assisted extraction was performed for 30 min at 40 kHz to enhance phytoconstituent release. The extract was filtered and centrifuged at 4000 rpm for 4 min. The supernatant was concentrated under reduced pressure using a rotary evaporator and stored at 4 °C for further analysis (Figure 2).

#### 2.2.2 LC-HRMS-based phytochemical validation

Phytochemical confirmation was performed using high-resolution liquid chromatography–electrospray ionization quadrupole time-of-flight mass spectrometry (LC-HRMS) on a Waters XEVO-G2-XS QTOF system. Separation was achieved using a reverse-phase C18 column under gradient elution conditions.

Since commercial reference standards were not employed, compound identification was based on high-resolution accurate mass measurement, isotope distribution pattern matching, and comparison with the known molecular weights reported in the IMPPAT database and PubChem repositories. External mass calibration was performed prior to analysis using the instrument’s standard calibration solution to ensure mass accuracy within ±5 ppm.

The presence of liquiritin (m/z 431.1349), licoflavone A (m/z 323.1284/321.1133), and glycyrol (m/z 369.1347) was confirmed based on their protonated molecular ions [M + H]^+^ and accurate mass matching with database values (Martins et al., 2016; Dhanasekaran et al., 2024; Kim et al., 2023). Due to the absence of authentic standards, this analysis was qualitative in nature and aimed at molecular confirmation rather than quantification.

#### 2.2.3 Fungal strains and inoculum preparation

Antifungal activity was evaluated against eight yeast isolates comprising one reference strain (*C. albicans* ATCC 10231) and seven environmental drug-resistant isolates including *C. albicans, C. krusei, C. rugosa, C. tropicalis, Cryptococcus randhawii, Trichosporon asahii,* and *Trichosporon dohaense.* Fresh fungal cultures were grown on Sabouraud Dextrose Agar (SDA) for 24 h at 35 °C ± 2 °C. Inoculum suspensions were prepared in sterile saline and adjusted to 0.5 McFarland turbidity standard, followed by dilution to achieve a final cell density of approximately 1–5 × 10^5^ CFU/mL for susceptibility testing (Smith and Kirby, 2018).

#### 2.2.4 Agar well diffusion assay (qualitative screening)

Preliminary antifungal screening was conducted using the agar well diffusion method as described by Erhonyota et al. (2023), with minor modifications (Erhonyota et al., 2023). The concentrated crude extract of *G. glabra* (10 g) was dissolved in 10 mL of 2% sterile dimethyl sulfoxide (DMSO) to prepare the stock solution. A 2% DMSO solution was employed to dissolve the concentrated crude extract owing to the inadequate water solubility of the phytochemicals. While DMSO concentrations beyond 1% may exhibit cytotoxicity in cell-based experiments, agar well diffusion studies allow for marginally higher concentrations due to the restricted diffusion of DMSO through the solid medium; however, the 2% DMSO control wells were compared as negative control to observe the comparative antagonist activity if any. The extract was serially diluted using a two-fold dilution method to obtain concentrations ranging from 100 to 1.56 mg/mL. Mueller Hinton Agar plates supplemented with the antibiotic chloramphenicol were prepared. Following the uniform distribution of the cultures, four wells of uniform diameter were created per plate at equal distances using a manual well borer. Plates were incubated at 35 °C ± 2 °C for 24–48 h, and zones of inhibition were measured in millimeters (Erhonyota et al., 2023).

This assay was used strictly as a qualitative screening tool to visualize antifungal activity, while quantitative potency was assessed using minimum inhibitory concentration (MIC) determination (Section 2.2.5).

#### 2.2.5 Determination of minimum inhibitory concentration (MIC) by broth microdilution

The minimum inhibitory concentration (MIC) of the *G. glabra* extract was determined using the broth microdilution method in sterile 96-well microtiter plates, following CLSI M27-A3 guidelines for yeasts with minor modifications in medium composition and inoculum volume (Smith and Kirby, 2018; Motsei et al., 2003).

Due to the poor aqueous solubility of the phytochemicals, the concentrated crude extract was initially dissolved in 2% (v/v) dimethyl sulfoxide (DMSO). Seven two-fold serial working concentrations of the extract were prepared ranging from 100, 50, 25, 12.5, 6.25, 3.125 to 1.56 mg/mL. For each test well, 100 µL of 2× Mueller–Hinton broth (2× MHB) and 100 µL of the respective working extract solution were added. Owing to this 1:1 dilution with broth, the final test concentrations in the wells were 50, 25, 12.5, 6.25, 3.125, 1.56, and 0.78 mg/mL, respectively. The eighth well served as the solvent control and contained 100 µL of 2× MHB and 100 µL of 2% DMSO, yielding a final DMSO concentration of 1% after mixing, but without any plant extract. An equal volume of the fungal inoculum was added to this well to assess any potential solvent-associated effect on fungal viability under broth microdilution conditions.

Fungal inoculum was prepared from 24-h-old cultures grown on SDA, suspended in sterile saline, and adjusted to a 0.5 McFarland standard (Smith and Kirby, 2018). A 5 µL aliquot of this suspension (≈500 CFU) was added to each well containing ∼200 µL of test mixture, resulting in a final inoculum density of approximately 2.4 × 10^3^ CFU/mL, which lies within the CLSI-recommended range (0.5–2.5 × 10^3^ CFU/mL) for MIC determination (Smith and Kirby, 2018; Motsei et al., 2003).

After incubation at 35 °C ± 2 °C for 24 h, 10 µL of resazurin dye solution (0.01% w/v) was added to each well as a colorimetric viability indicator, followed by further incubation for 2–4 h. The MIC was defined as the lowest final extract concentration that prevented the color change of resazurin from blue to pink. All experiments were performed in triplicate, and mean MIC values were used for analysis.

## 3 Results

### 3.1 Molecular docking analysis of phytochemicals against ERG11 and CDR2

**FIGURE 3:**
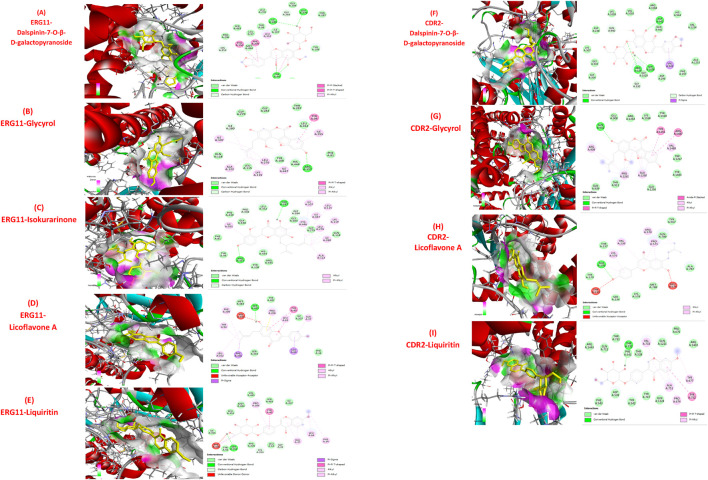
3D and 2D Molecular Docking visualization of potential phytochemicals “Dalspinin-7-O-β-D-galactopyranoside, Glycyrol, Isokurarinone, Licoflavone A and Liquirin” with target protein ERG11p **(A–E)** along with CDR2p **(F–I)**.

A molecular docking investigation of 17,967 phytochemicals targeting ERG11 revealed 11 prime candidates with robust binding affinities. Isocorilagin had the greatest binding energy (−10.8 kcal/mol), succeeded by Toddasin (−10.1 kcal/mol) and other compounds (Table 3), all surpassing the control drug fluconazole (−7.3 kcal/mol) and miconazole (−9.1 kcal/mol), as seen in Table 5. Five compounds—dalspinin-7-O-β-D-galactopyranoside (−9.5 kcal/mol), glycyrol (−9.4 kcal/mol), isokurarinone (−9.3 kcal/mol), licoflavone A (−9.3 kcal/mol), and liquiritin (−9.2 kcal/mol) satisfied the drug-likeness criteria established by Lipinski and Pfizer. Their predicted interactions with ERG11p are illustrated in Figures 3A–E, Table 3, highlighting key molecular contacts. To evaluate their dual-target potential, these five phytochemicals were further docked against the efflux pump protein CDR2. With the exception of Isokurarinone, the other four compounds exhibited robust binding to the CDR2 active site residues (binding energies: -8.5 to −9.5 kcal/mol), as seen in Figures 3F–I, Table 4. The observed interaction patterns suggest potential inhibition of drug efflux activity. Based on their superior docking performance against ERG11 and CDR2, the five phytochemicals and ERG11 protein complexes as well as four CDR2 complexes were proposed for MD simulation to examine the temporal consistency of the complex’s interactions within an optimal system.

**TABLE 3:** Binding energies of top 11 phytocompounds interacting with ERG11 active site residues.

Sr. No.	Phytochemical name	BE	Active site residues forming H bonds with leading phytocompounds	nDHB	nAHB
1	Isocorilagin	−10.8	SER354, HIS444, CYS446, ILE447	2	2
2	Toddasin	−10.1	TYR94	0	1
3	Dalspinin-7-O-β-D-galactopyranoside	−9.5	TYR94, TYR94, GLY279, ILE280	2	2
4	Glycyrol	−9.4	ARG357	0	1
5	Javanicinoside C	−9.4	TYR94, PHE439	2	0
6	Isokurarinone	−9.3	THR287, ARG357	1	1
7	Licoflavone A	−9.3	SER483	1	0
8	Thalrugosaminine	−9.3	TYR108, THR287	0	2
9	Liquiritin	−9.2	SER354	1	0
10	Kaempferol 3-neohesperidoside	−9.2	TYR108, MET484	2	0
11	Ipobscurine B	−8.6	MET484	1	0

BE, Binding Energy (kcal/mol), nDHB, number of Donating Hydrogen bonds; nAHB, number of Accepting Hydrogen bonds.

**TABLE 4:** Binding energies of four leading phytocompounds interacting with CDR2 active site residues.

Sr. No.	Phytochemical name	BE	Active site residues forming H bonds with leading phytocompounds	nDHB	nAHB
3	Dalspinin-7-O-β-D-galactopyranoside	−8.5	ASP1102, PRO1103, TRP1113	2	2
4	Glycyrol	−8.6	TRP1113	0	2
7	Licoflavone A	−9.0	NIL	0	0
9	Liquiritin	−9.5	ARG64	0	1

BE, Binding Energy (kcal/mol), nDHB, number of Donating Hydrogen bonds; nAHB, number of Accepting Hydrogen bonds.

#### 3.1.1 Validation of docking protocol using reference control drugs

To validate the reliability and biological relevance of the docking protocol, established antifungal control drugs were docked against their respective targets. Miconazole and fluconazole, well-known ERG11 inhibitors (Francois et al., 2006), exhibited binding energies of −9.1 kcal/mol and −7.3 kcal/mol, respectively (Table 5). Both drugs occupied the canonical catalytic pocket of ERG11 and interacted with key active-site residues such as TYR108, HIS444, ILE280, and CYS446, which are known to be critical for azole binding and enzymatic inhibition.

**TABLE 5:** Binding energies and interacting residues of reference control drugs docked against ERG11 and CDR2.

Sr. No.	Control drug-target docking	BE	Interacting active site residues with the drug	
1	Miconazole-ERG11	−9.1	Carbon hydrogen bonds	TYR108, ILE280
			Alkyl bonds	ILE107, LEU115, LYS119, PHE204, LEU276, VAL485
			Van der waals	PHE102, GLN118, LEU126, GLY279, GLY283, GLY284, HIS286, THR287, LEU352, HIS444, MET484, CYS446, ILE447, GLY448
2	Fluconazole-ERG11	−7.3	Conventional hydrogen bonds	TYR108, ARG445
			Carbon hydrogen bond	HIS444
			Halogen (fluorine) bond	GLN118
			Alkyl bonds	ILE107, LYS119, ILE280
			Van der waals	THR98, PHE102, LEU115, ALA122, PHE204, LEU276, GLY283, CYS446, ILE447
3	Beauvericin-CDR2	−12.2	Alkyl bonds	ARG570, PRO571
			Van der waals	VAL239, HIS240, PRO242, ASN295, PHE297, TYR567, GLU568, ILE572, THR650, ILE651, VAL994, PRO1246, TYR1247, LYS1250, ILE1341

Additionally, beauvericin, a documented modulator of fungal ABC transporters (Tong et al., 2016), showed strong binding to CDR2 (−12.2 kcal/mol) and formed extensive hydrophobic and van der Waals interactions with residues lining the transmembrane drug-transport channel. The interaction profiles and binding energies of these control ligands were consistent with previously reported experimental and computational studies, thereby confirming the robustness and predictive reliability of the docking workflow. Importantly, several of the screened phytochemicals exhibited binding energies comparable to or stronger than those of the reference drugs, supporting their potential as effective dual inhibitors of ERG11 and CDR2.

### 3.2 Physicochemical characteristics of selected lead phytochemicals

The physicochemical properties of the 11 top-scoring phytochemicals were analyzed to evaluate their structural suitability for drug-like behavior (Table 6). These compounds belonged predominantly to the flavonoid, flavonoid glycoside, coumarin, ellagitannin, and alkaloid classes, reflecting substantial chemical diversity within the screened library. Among the five prioritized lead compounds, licoflavone A (322.36 Da) and glycyrol (366.37 Da) fell well within the optimal molecular weight range for oral drug candidates, whereas isokurarinone (438.52 Da) remained moderately compliant. In contrast, dalspinin-7-O-β-D-galactopyranoside (490.42 Da) and liquiritin (418.41 Da), owing to their glycosidic nature, exhibited higher molecular weights and elevated hydrogen bond donor/acceptor counts.

**TABLE 6:** Physicochemical properties of selected lead compounds.

Phytochemicals	Phytocompound category	IMPPAT ID	Ch. formula	M. wt.	nHBD	nHBA
Isocorilagin	Ellagitannin	IMPHY011099	C_27_H_22_O_18_	634.45	11	18
Toddasin	Coumarin	IMPHY001272	C_32_H_30_O_10_	544.21	0	8
Dalspinin-7-O-β-D-galactopyranoside	Flavonoid glycoside	IMPHY012841	C_23_H_22_O_12_	490.42	5	12
Glycyrol	Coumarin	IMPHY004405	C_21_H_18_O_6_	366.37	2	6
Javanicinoside C	Flavonoid glycoside	IMPHY008323	C_27_H_36_O_11_	538.59	4	11
Isokurarinone	Flavonoid	IMPHY012797	C_26_H_30_O_6_	438.52	3	6
Licoflavone A	Flavonoid	IMPHY004395	C_20_H_18_O_4_	322.36	2	4
Thalrugosaminine	Benzylisoquinoline alkaloid	IMPHY010935	C_39_H_44_N2O_7_	652.79	0	9
Liquiritin	Flavonoid glycoside	IMPHY005055	C_21_H_22_O_9_	418.41	5	9
Kaempferol 3-neohesperidoside	Flavonoid glycoside	IMPHY014967	C_27_H_30_O_15_	594.52	9	15
Ipobscurine B	Indole alkaloid	IMPHY005020	C_33_H_52_O_9_	518.57	6	7

Ch. Formula, chemical formula, M. wt., molecular weight, nHBD, number of Hydrogen bond donors; nHBA, number of Hydrogen bond acceptors.

Hydrogen bonding capacity varied markedly among ligands, with liquiritin (5 HBD, 9 HBA) and dalspinin-7-O-β-D-galactopyranoside (5 HBD, 12 HBA) demonstrating extensive polar functionality, which favors target binding but may compromise passive membrane permeability. In contrast, licoflavone A (2 HBD, 4 HBA) exhibited a balanced polarity profile consistent with favorable permeability. Collectively, the physicochemical analysis revealed that aglycone flavonoids (licoflavone A and isokurarinone) possess superior drug-like structural characteristics compared to glycosidic derivatives, which, although highly interactive, may be limited by permeability constraints.

### 3.3 Drug-likeness, bioavailability, and ADMET analysis

**FIGURE 4:**
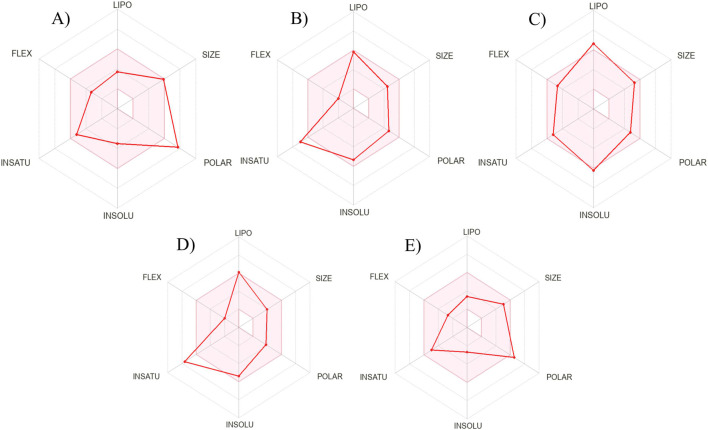
Bioavailability radar analysis of **(A)** Dalspinin-7-O-β-D-galactopyranoside, **(B)** Glycyrol, **(C)** Isokurarinone, **(D)** Licoflavone **(A, E)** Liquiritin.

The drug-likeness and pharmacokinetic profiles of the five lead compounds were assessed using SwissADME and ADMETlab predictions (Table 7; Figure 4). All five compounds adhered to the Lipinski Rule of Five, demonstrating fundamental oral drug-likeness compliance. Significant diversity was noted with sophisticated pharmacokinetic filters. Isokurarinone exhibited the highest overall drug-likeness score (QEDw = 0.702), high gastrointestinal absorption, and the ability to cross the blood–brain barrier, reflecting excellent membrane permeability. However, its poor predicted aqueous solubility suggests that formulation optimization would be required for systemic delivery. Conversely, dalspinin-7-O-β-D-galactopyranoside displayed high solubility but poor gastrointestinal absorption, reflecting the classic solubility–permeability trade-off frequently observed in glycosylated flavonoids.

**TABLE 7:** Drug likeness profile of selected phytochemicals.

Phytochemical	Dalspinin-7-O-β-D-galactopyranoside	Glycyrol	Lycoflavone A	Isokurarinone	Liquiritin
Lipinski rule of 5 filter	Passed	Passed	Passed	Passed	Passed
Pfizer 3/75 filter	Passed	Failed	Failed	Failed	Passed
Veber filter	Failed	Passed	Passed	Passed	Passed
Ghose filter	Failed	Passed	Failed	Passed	Passed
Weighted quantitative estimate of drug likeness (QEDw) score	0.3259	0.4059	0.4864	0.702	0.4594
Bioavailability score	0.55	0.55	0.55	0.55	0.55
Solubility class [ESOL]	Soluble	Moderately soluble	Poorly soluble	Moderately soluble	Soluble
Solubility class [Silicos-IT]	Soluble	Poorly soluble	Moderately soluble	Poorly soluble	Soluble
Blood brain barrier permeation	No	No	No	Yes	No
Gastro intestinal absorption	Low	High	High	High	Low

Liquiritin and glycyrol exhibited a well-rounded ADMET profile, marked by moderate solubility, satisfactory bioavailability scores (0.55), advantageous gastrointestinal absorption, and lack of blood–brain barrier penetration, which mitigates the risk of central nervous system toxicity. Licoflavone A, despite its low solubility, satisfied several drug-likeness criteria and had promising permeability potential. The failure of certain compounds in the Pfizer 3/75 and Ghose filters signifies significant safety and metabolic risks, highlighting that a high binding affinity alone is inadequate for lead qualification. These findings underscore the necessity of considering pharmacokinetic and solubility limitations in conjunction with molecular docking efficacy when evaluating antifungal candidates.

### 3.4 Molecular dynamic (MD) simulation

**FIGURE 5:**
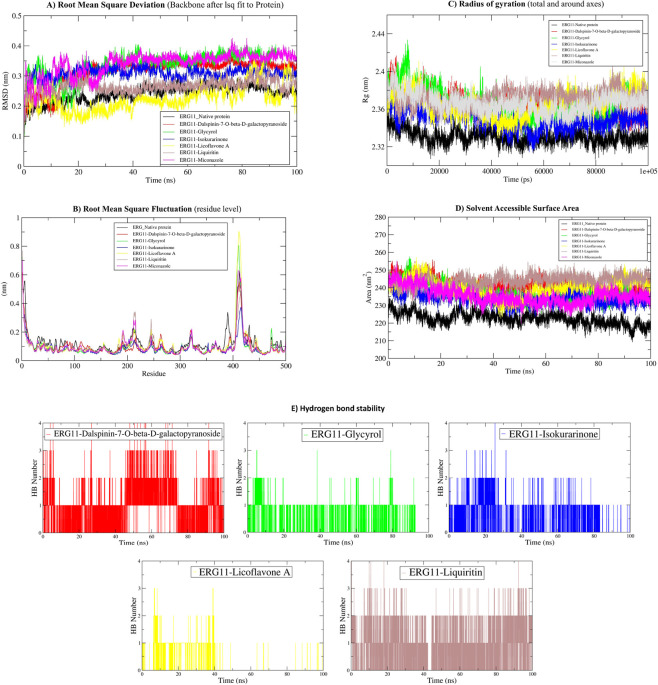
Molecular Dynamic (MD) simulations of apoprotein and complex form of ERG11p with leading phytochemical ligands. **(A)** Root Mean Square Deviation (backbone after lsk fit to protein), **(B)** Root Mean Square Fluctuation (residue level), **(C)** Radius of gyration (total and around axes), **(D)** Solvent Accessible Surface Area, **(E)** Hydrogen bond stability. RMSD = Root Mean Square Deviation, RMSF = Root Mean Square Fluctuation, SASA = Solvent Accessible Surface Area, Rg = Radius of Gyration, HB = Hydrogen bond. Note: Black = apoprotein ERG11p, Red = Dalspinin-7-O-beta-D-galactopyranoside, Green = Glycyrol, Blue = Isokurarinone, Yellow = Licoflavone A, Brown = Liquirin, and Magenta = Miconazole (no H bond interaction).

Molecular Dynamics (MD) simulations were performed for 100 ns to assess the structural and dynamic impacts of five phytochemicals and the conventional antifungal agent miconazole on the *Candida albicans* ERG11 protein. Post-simulation analysis encompassed RMSD, RMSF, Rg, SASA, and H-bond profiling (Figure 5). The apo-ERG11 protein exhibited a low RMSD (∼0.20 nm), a compact Rg of around 2.32 nm, and a stable SASA ranging from 225 to 230 nm^2^, signifying a stable, folded conformation during the simulation.

Ligand binding elicited varied degrees of conformational alteration. Glycyrol and liquiritin demonstrated the most significant deviations, with RMSD values reaching around 0.42 nm, heightened Rg (2.40–2.43 nm), and enhanced SASA (>250 nm^2^). RMSF study indicated significant flexibility around residue ∼420, with oscillations of up to 0.80 nm. Liquiritin consistently exhibited 1–4 hydrogen bonds during the 100 ns trajectory, whereas glycyrol occasionally produced 1–2, indicating high to moderate binding stability. The detected structural destabilization, along with persistent anchoring, suggests a possibility of impairing ERG11 function.

Dalspinin-7-O-β-D-galactopyranoside and licoflavone A induced intermediate destabilization. RMSD values varied between 0.24 and 0.30 nm, Rg ranged from 2.36 to 2.39 nm, while SASA exhibited moderate increases. Dalspinin-7-O-β-D-galactopyranoside established up to four hydrogen bonds with dynamic stability, but the interactions of licoflavone A reduced after around 30 nanoseconds, indicating a poorer long-term affinity.

Isokurarinone elicited little structural alteration (RMSD ∼0.22–0.24 nm, R_g_ ∼2.33–2.35 nm), characterized by low RMSF and infrequent, transitory hydrogen bonding (1–2 bonds intermittently). These characteristics indicate restricted inhibitory capacity owing to inadequate conformational alteration.

Miconazole, serving as a reference, preserved ERG11 in a rather compact conformation, with RMSD and Rg values akin to the apo form, and significantly did not establish conventional hydrogen bonds throughout the simulation. Conversely, many phytochemicals—specifically liquiritin, glycyrol, and dalspinin-7-O-β-D-galactopyranoside—exhibited prolonged hydrogen bonding and promoted enhanced conformational flexibility compared to miconazole, indicating the possibility of attaining inhibitory effects via alternative binding pathways.

These results indicate that certain phytochemicals can modulate ERG11 dynamics more efficiently than miconazole, while preserving stable connections. This substantiates their evaluation as prospective lead candidates for the development of alternative antifungal treatments targeting *Candida* infections, necessitating additional *in-vitro* and *in-vivo* validation.

### 3.5 Mass spectrometric detection of liquiritin, licoflavone A, and glycyrol

**FIGURE 6:**
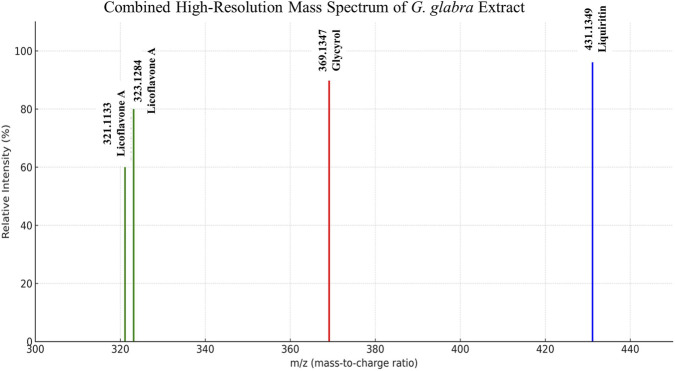
High-resolution TOF MS (ES^+^) spectrum of *Glycyrrhiza glabra* extract exhibiting [M + H]^+^ peaks of liquiritin (m/z 431.1349), licoflavone A (m/z 323.1284, 321.1133), and glycyrol (m/z 369.1347), indicating their existence in the sample.

High-resolution electrospray ionization time-of-flight mass spectrometry (TOF MS ES^+^) was utilized to verify the existence of three bioactive compounds—liquiritin (MW 430.41 Da), licoflavone A (MW 322.36 Da), and glycyrol (MW 366.37 Da)—in the extract of *G. glabra*. The combined spectrum (Figure 6) exhibits distinctive [M + H]^+^ peaks for liquiritin at m/z 431.1349, licoflavone A at m/z 323.1284 and 321.1133, and glycyrol at m/z 369.1347. These results align with the anticipated protonated molecule ions, with slight discrepancies remaining within acceptable experimental margins of error. Collectively, these spectrum characteristics validate the concurrent existence of all three intended phytoconstituents in the examined extract.

**FIGURE 7:**
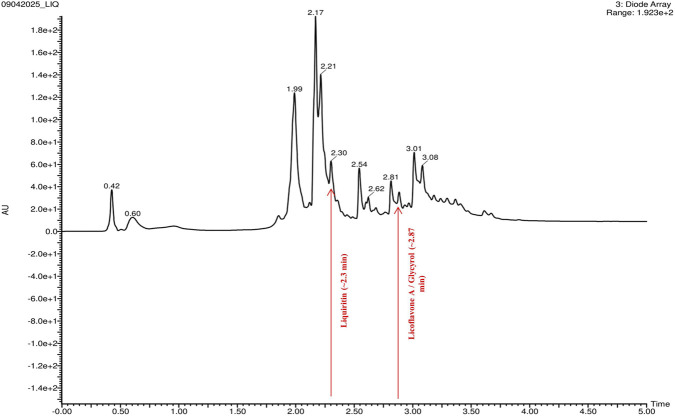
Chromatographic profile of *Glycyrrhiza glabra* extract acquired using diode array detection (DAD), demonstrating retention of chosen bioactive phytocomponents.

The chromatographic profile of the *G. glabra* extract (Figure 7), obtained using diode array detection (DAD), displayed numerous peaks within a 2–3.5 min interval, indicating the presence of several phytoconstituents. A distinct and well-defined peak at roughly 2.30 min corresponds to liquiritin, verified by a distinctive [M + H]^+^ ion at m/z 431.1349 in the high-resolution TOF mass spectrum. Furthermore, two overlapping peaks identified between 2.81 and 3.08 min indicate the co-elution of licoflavone A and glycyrol, affirmed by the corresponding molecular ions found at m/z 323.1284/321.1133 and m/z 369.1347. The retention behavior and signal intensities align with the physicochemical characteristics of these flavonoid molecules. The chromatographic and spectrum results together confirm the existence of these three bioactive compounds in the extract of *G. glabra.*

### 3.6 *In Vitro* evaluation of the antifungal activity

The antifungal effectiveness of *G*. *glabra* root extract, which includes liquiritin, licoflavone A, and glycyrol, was assessed against eight drug-resistant yeast isolates utilizing the agar well diffusion technique (Erhonyota et al., 2023). The test panel comprised one reference strain (*Candida albicans* ATCC10231) and seven environmental isolates: *C. albicans, C. krusei, C. rugosa, C. tropicalis, Cryptococcus randhawii, Trichosporon asahii,* and *Trichosporon dohaense*.

The extract was evaluated at seven two-fold serial dilutions, spanning from 100 mg/mL to 1.56 mg/mL. The concentrated extract was entirely dissolved in 2% DMSO to guarantee total solubility, and an identical 2% DMSO solution, devoid of extract, served as the negative control in a separate well.

**FIGURE 8:**
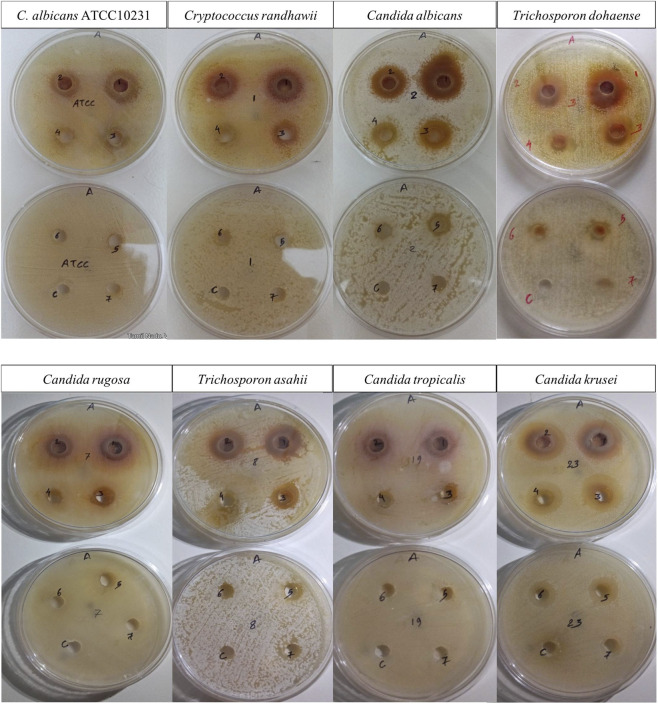
*In-vitro* confirmation of antifungal activity of two-fold serially diluted *Glycyrrhiza glabra* extract containing liquiritin, licoflavone A, and glycyrol. Well one contains 100 mg/mL, followed by wells 2–7 with 50, 25, 12.5, 6.25, 3.125, and 1.56 mg/mL, respectively. The well labeled C represents the solvent control and shows no antifungal activity. Zones of inhibition indicate concentration-dependent antifungal activity.

A concentration-dependent inhibitory effect was seen in all isolates. At the maximum concentration (100 mg/mL), inhibition zones varied from 19 mm (*C. rugosa*) to 25 mm (*C. albicans*, environmental isolate), whereas at the minimum dose (1.56 mg/mL), only *C. krusei* exhibited a detectable inhibition of 10 mm. Among the environmental isolates, *C. albicans*, *C. krusei*, and *T. dohaense* exhibited the greatest susceptibility, sustaining measurable inhibition at reduced doses. The *C. albicans* ATCC10231 strain exhibited moderate susceptibility, with inhibition zones diminishing from 20 mm at 100 mg/mL to a total loss of activity at 6.25 mg/mL (Motsei et al., 2003) (Figure 8).

No inhibition was noted in the control wells with 2% DMSO alone, so indicating that the antifungal action was attributable to the phytochemicals liquiritin, licoflavone A, and glycyrol. The comprehensive inhibitory zone statistics are displayed in Table 8.

**TABLE 8:** Size of zone of inhibition of selected phytochemicals against drug resistant yeasts pathogens.

Isolate no. (Plate No.)	100 mg/mL	50 mg/mL	25 mg/mL	12.5 mg/mL	6.25 mg/mL	3.125 mg/mL	1.56 mg/mL	Control
ATCC (*Candida albicans* ATCC10231)	20	16	14	8	0	0	0	0 mm
1 (*Cryptococcus randhawii*)	20	18	14	12	0	0	0	0 mm
2 (*Candida albicans*)	25	20	18	14	12	6	0	0 mm
3 (*Trichosporon dohaense*)	23	20	18	10	8	6	0	0 mm
7 (*Candida rugosa*)	19	17	12	0	0	0	0	0 mm
8 (*Trichosporon asahii*)	21	18	14	12	8	0	0	0 mm
19 (*Candida tropicalis*)	20	18	12	8	0	0	0	0 mm
23 (*Candida krusei*)	24	24	22	20	14	12	10	0 mm

### 3.7 Minimum inhibitory concentration (MIC) determination

The antifungal potency of the *G. glabra* extract was quantitatively assessed using a resazurin-based broth microdilution assay. Seven two-fold serial concentrations (final well concentrations: 50, 25, 12.5, 6.25, 3.125, 1.56, and 0.78 mg/mL) were tested against nine yeast isolates, including *C. albicans* ATCC 10231 and eight drug-resistant environmental strains. The eighth well in each row served as the solvent control and contained 1% final DMSO without plant extract along with the same inoculum volume, confirming that the solvent did not inhibit fungal growth.

**FIGURE 9:**
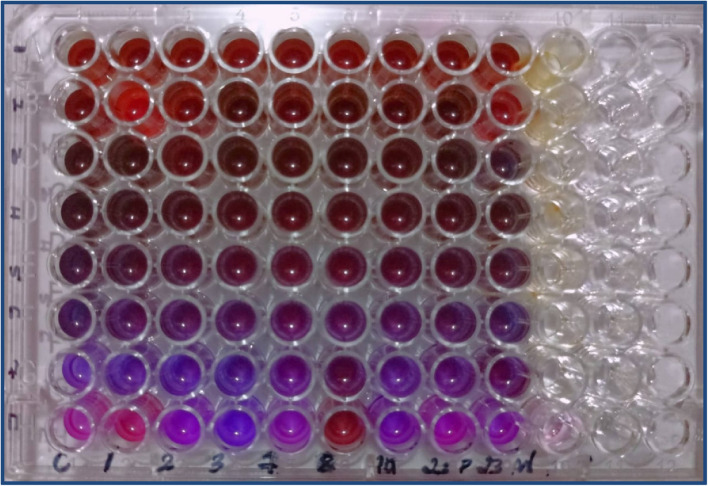
Resazurin-based microbroth dilution assay showing MIC determination of *Glycyrrhiza glabra* extract against nine yeast isolates.

At the lowest concentrations (≤1.56 mg/mL), wells showed a pink-to-purple color, indicating active metabolic reduction of resazurin and therefore viable fungal growth. Beginning at 3.125 mg/mL, all isolates showed a complete absence of pink coloration, with wells retaining a stable blue/purple color, signifying full inhibition of fungal metabolic activity. Higher concentrations (6.25–50 mg/mL) also showed complete inhibition as expected (Figure 9).

A reddish or orange tint observed in the highest concentration wells (50–25 mg/mL) was attributed to the intrinsic pigmentation of the crude extract and not to metabolic activity; these wells did not exhibit the characteristic pink resorufin color associated with viable growth.

Based on the lowest concentration that prevented any visible color change from resazurin to resorufin, the MIC for the *G. glabra* extract was determined to be 3.125 mg/mL for all nine isolates, indicating a consistent and broad-spectrum inhibitory effect.

Seven two-fold serial concentrations of the extract (final well concentrations: 50, 25, 12.5, 6.25, 3.125, 1.56, and 0.78 mg/mL) were tested in each row. The eighth well served as the solvent control containing 1% DMSO without extract. Wells exhibiting pink coloration indicate viable fungal growth due to metabolic reduction of resazurin to resorufin, whereas purple/blue wells indicate growth inhibition. Complete inhibition was consistently observed at 3.125 mg/mL, which was therefore recorded as the MIC for all nine isolates. The reddish tint at the highest concentrations (50–25 mg/mL) reflects the intrinsic pigmentation of the crude extract and not metabolic activity (Figure 9).

### 3.8 Correlation between *in-silico* binding affinity and *in-vitro* antifungal activity

A qualitative correlation analysis was performed to evaluate the relationship between computational predictions and experimental results, specifically analyzing the ERG11 binding energies of *G. glabra*-derived phytochemicals (liquiritin, glycyrol, and licoflavone A) in relation to the experimentally determined minimum inhibitory concentration (MIC) of the plant extract. The three compounds exhibited substantial docking affinities (−9.2 to −9.5 kcal/mol) and sustained interactions in molecular dynamics simulations, corresponding with the extract’s stable minimum inhibitory concentration of 3.125 mg/mL across all evaluated isolates (Rocha et al., 2019; Srivastava et al., 2022; Li et al., 2021).

The association between elevated projected binding affinity and significant growth inhibition suggests that these ERG11-binding phytochemicals likely play a role in the antifungal efficacy of the extract. Minor disparities in computational potency and extract MIC may be attributed to the synergistic effects of phytochemicals, changes in solubility, and matrix impacts inherent to crude plant extracts (Singh et al., 2016; Martins et al., 2016).

The results demonstrate a satisfactory agreement between *in silico* and *in vitro* data, supporting the notion that *G. glabra* phytochemicals have antifungal activity, partially through the inhibition of ERG11, consistent with docking and molecular dynamics modeling studies.

## 4 Discussion

The present study integrates *in silico* screening, molecular dynamics simulation, phytochemical confirmation, and *in vitro* validation to identify Indian medicinal phytochemicals with dual inhibitory potential against ERG11 and CDR2 in drug-resistant *C.albicans*. Unlike conventional studies that focus on a single molecular target, the dual-target strategy adopted here is particularly relevant in the context of azole resistance, which commonly arises from simultaneous ERG11 mutation/overexpression and CDR2-mediated drug efflux (Cowen et al., 2015; Prasad and Rawal, 2014; White et al., 2002). Our findings strongly support the feasibility of plant-derived flavonoids and coumarins as multi-target antifungal agents.

### 4.1 Correlation of docking and MD results with existing ERG11 inhibition studies

ERG11 (lanosterol 14-α-demethylase) is a cytochrome P450 enzyme essential for ergosterol biosynthesis, and its inhibition leads to membrane destabilization and fungal cell death (Lepesheva and Waterman, 2007). Azole resistance is largely driven by ERG11 overexpression, point mutations, and altered sterol composition (Flowers et al., 2015; Dunkel et al., 2008; Singh et al., 2016). In the present study, all five lead phytochemicals showed significantly stronger binding affinity to ERG11 (−9.2 to −9.5 kcal/mol) than fluconazole (−7.3 kcal/mol), indicating a potentially superior inhibitory profile.

Flavonoids such as licoflavone A and liquiritin belong to a class of polyphenols previously reported to inhibit fungal sterol biosynthesis by interacting with cytochrome P450 enzymes (Martins et al., 2016; Zang, 2020). Earlier reports demonstrate that licorice-derived flavonoids suppress ergosterol synthesis and increase membrane permeability in *Candida* spp., thereby enhancing antifungal susceptibility (Singh et al., 2016; Martins et al., 2016). The stable hydrogen bonding and increased RMSD, Rg, and SASA values observed for liquiritin and glycyrol in MD simulations in our study confirm their ability to induce sustained conformational destabilization of ERG11, a prerequisite for effective enzymatic inhibition.

Unlike miconazole, which showed minimal hydrogen bonding and maintained ERG11 in a compact state, liquiritin and glycyrol formed persistent hydrogen bonds (1–4 bonds throughout 100 ns) and promoted higher protein flexibility, suggesting a distinct and possibly resistance-evading binding mechanism. Similar non-azole binding modes disrupting ERG11 dynamics have been reported for terpenoids and flavonoids in resistant *Candida* strains (Domingos and Batzelladine, 2024; Dantas et al., 2025; Houshmandzad et al., 2022).

### 4.2 Dual inhibitory action on ERG11 and CDR2: mechanistic implications

CDR2 is an ATP-binding cassette (ABC) transporter that actively pumps azole drugs out of fungal cells and is a major contributor to multidrug resistance in *C. albicans* (Sanglard et al., 1999). The four phytochemicals—dalspinin-7-O-β-D-galactopyranoside, isokurarinone, glycyrol, licoflavone A, and liquiritin demonstrated strong binding affinities toward CDR2 (−8.5 to −9.5 kcal/mol), with liquiritin showing the highest CDR2 binding (−9.5 kcal/mol).

The ability of these compounds to interact with key residues such as ARG64, TRP1113, and ASP1102 suggests potential obstruction of the drug-transport channel, thereby reducing azole efflux. This dual activity is pharmacologically important because ERG11 inhibition blocks ergosterol synthesis, weakening the fungal membrane. CDR2 inhibition prevents antifungal drug extrusion, restoring intracellular drug accumulation.

Previous studies on flavonoids such as quercetin and kaempferol have shown efflux pump inhibition in resistant *Candida* strains, leading to restored azole sensitivity (Prasad and Rawal, 2014; Prasad et al., 2015; Sharma et al., 2025). Our findings extend this mechanism to licoflavone A, glycyrol, and liquiritin, thereby offering a molecular explanation for the strong *in-vitro* antifungal activity observed against resistant isolates. In contrast, isokurarinone lacked binding to CDR2 and showed minimal conformational perturbation of ERG11, which rationally explains its comparatively weaker antifungal potential despite good drug-likeness parameters.

Phytochemicals like flavonoids are also known to inhibit efflux pumps and potentiate azole activity. The strong CDR2 binding observed for liquiritin, glycyrol, and licoflavone A supports this mechanism. The uniform MIC across all isolates additionally suggests synergistic interactions within the phytochemical mixture, consistent with reports of cooperative antifungal actions among flavonoids (Singh et al., 2016; Martins et al., 2016).

### 4.3 Biological justification of the selected fungal species in relation to ERG11 and CDR2

The fungal strains evaluated in this study include drug-resistant *C. albicans, C. krusei, C. tropicalis, C. rugosa, Cryptococcus randhawii,* and *Trichosporon* spp. These clinically relevant yeasts were isolated from environments contaminated with human waste and bird excreta. They are known to exhibit ERG11-and efflux pump–mediated resistance mechanisms (Arendrup and Patterson, 2017; Arendrup, 2014; Pfaller and Diekema, 2007; Subramanian and Mathai, 2005; Shourian and Qureshi, 2019). Specifically *C. albicans* and *C. tropicalis* overexpress CDR1/CDR2 transporters under azole pressure (Vermitsky and Edlind, 2004). *C. krusei* is intrinsically fluconazole-resistant due to reduced ERG11 affinity (Rex et al., 1995). *Cryptococcus* and *Trichosporon* species contain functional ERG11 homologs and ABC transporters, contributing to reduced azole susceptibility (Perfect et al., 2010; Colombo et al., 2011).

Therefore, the strong antifungal activity observed for the *G. glabra* extract (confirmed to contain glycyrol, licoflavone A, and liquiritin by HR-LCMS) against this diverse fungal panel directly correlates with the dual molecular targeting of ERG11 and CDR2 demonstrated *in silico*. This mechanistic alignment between computational prediction and experimental antifungal outcomes substantially strengthens the biological relevance of the proposed drug candidates.

### 4.4 Role of flavonoids and coumarins as multi-target antifungal agents

The majority of lead compounds identified belong to flavonoid and coumarin classes, which are known for antifungal properties including membrane disruption, ROS generation, efflux pump inhibition, and synergy with azoles (Cushnie and Lamb, 2005; de Freitas et al., 2019; Rocha et al., 2019; Srivastava et al., 2022). Compounds such as liquiritin and licoflavone A, derived from *G. glabra*, have previously been reported to exhibit broad-spectrum antimicrobial and anti-biofilm activity (Mamedov and Egamberdieva, 2019), but their specific molecular interactions with ERG11 and CDR2 were previously uncharacterized. Our study provides the first structure-based mechanistic validation of their dual-target antifungal action. Furthermore, the persistent hydrogen bonding and enhanced protein flexibility observed in MD simulations for liquiritin and glycyrol clearly indicate long-term inhibitory stability, a crucial requirement for resistance-breaking antifungal therapeutics.

Observed inconsistencies—such as strong ERG11 docking but limited solubility for licoflavone A—reflect the pharmacokinetic limitations commonly associated with natural flavonoids (Rocha et al., 2019; Srivastava et al., 2022; Li et al., 2021). Additionally, the failure of certain compounds to meet Veber or medicinal chemistry filters suggests possible permeability or toxicity liabilities, indicating a need for formulation or structural optimization. Comparable dual in silico–in vitro antifungal mechanisms were recently demonstrated for thymoquinone, which inhibited virulence pathways and biofilm formation in *C. albicans* through combined molecular docking and experimental validation (Khan et al., 2024).

### 4.5 Clinical significance, novelty, and therapeutic implications

Current antifungal therapy is challenged by rising azole resistance, toxicity of polyenes, and high cost of echinocandins (Perlin, 2015). The phytochemicals identified in this study combine strong ERG11 inhibition, efflux pump interference, favorable drug-likeness, and broad-spectrum antifungal activity. This multi-mechanistic profile distinguishes them from existing azoles (ERG11-specific) and efflux inhibitors such as beauvericin (Tong et al., 2016).

#### 4.5.1 Future directions include

##### 4.5.1.1 Nanoparticle/liposomal formulations to enhance solubility and permeability of flavonoids

Nanoparticle-mediated enhancement of antifungal phytochemicals has been shown to significantly improve therapeutic outcomes, as demonstrated by liposomal thymoquinone achieving potent activity against fluconazole-resistant *C. albicans in vivo* (Khan et al., 2015). This supports the feasibility of nanocarrier formulation for *G. glabra*–derived compounds.

##### 4.5.1.2 Isolation and SAR studies to quantify the individual and synergistic contributions of phytochemicals

Purification of liquiritin, glycyrol, and licoflavone A will enable determination of individual MIC values, facilitating structure–activity relationship (SAR) analysis. Understanding synergistic versus additive interactions will clarify whether the strong *in vitro* potency of the crude extract results from multi-compound synergy or a dominant active molecule (Rocha et al., 2019; Srivastava et al., 2022; Li et al., 2021).

##### 4.5.1.3 *In vivo* studies to determine toxicity, pharmacokinetics, and therapeutic efficacy

Rodent models of candidiasis should be employed to evaluate systemic distribution, host toxicity, immunomodulatory effects, and therapeutic performance. These data are essential for determining whether the phytochemicals retain potency under physiological conditions and for establishing safe therapeutic margins.

##### 4.5.1.4 Medicinal chemistry optimization to improve permeability and reduce metabolic liabilities

Structural modification of flavonoids—such as deglycosylation, methylation, or prodrug design—may enhance membrane permeability, metabolic stability, and bioavailability (Thompson, 2001). Rational modification guided by docking, ADMET predictions, and MD simulations could yield more potent and drug-like derivatives suitable for preclinical development (Di and Kerns, 2015).

Overall, the findings support *G. glabra* phytochemicals as novel dual-target antifungal leads with significant translational potential against drug-resistant *Candida* species.

## 5 Conclusion

This work offers extensive mechanistic and experimental data corroborating the antifungal efficacy of certain phytochemicals derived from *G. glabra* and several Indian medicinal herbs. Liquiritin, glycyrol, and licoflavone A, identified in *G. glabra* extracts, exhibited *in-vitro* growth inhibition against drug-resistant *Candida* and non-*Candida* yeasts, and displayed more enduring and structurally disruptive interactions with ERG11 compared to miconazole in molecular dynamics simulations. Dalspinin-7-O-β-D-galactopyranoside and isokurarinone also exhibited advantageous computational profiles, and *in-vitro* experimental confirmation is advised to substantiate their antifungal efficacy.

This study integrates *in silico* screening, molecular dynamics, phytochemical extraction, molecule verification, and biological tests, therefore connecting ancient medicinal expertise with contemporary antifungal discovery processes. Future endeavors must prioritize *in vivo* effectiveness assessments, pharmacokinetic refinement, and structure–activity relationship (SAR) investigations to promote the conversion of these natural compounds into clinically applicable antifungal medicines.

## Data Availability

The original contributions presented in the study are included in the article/supplementary material, further inquiries can be directed to the corresponding author.
